# An interactive multi-entry key to the species of *Megalostomis* Chevrolat, with description of a new species from Paraguay (Chrysomelidae, Cryptocephalinae)

**DOI:** 10.3897/zookeys.425.7631

**Published:** 2014-07-10

**Authors:** Federico A. Agrain

**Affiliations:** 1 Laboratorio de Entomología, Instituto Argentino de Investigaciones de las Zonas Áridas (IADIZA, CCT-CONICET Mendoza), C.C. 507, 5500 Mendoza, Argentina

**Keywords:** Lucid interactive key, Clytrini, Megalostomina, new species, new records

## Abstract

The main goal of this contribution is to release an interactive multi-entry key to all known species of the genus *Megalostomis* Chevrolat. This key constitutes a new tool created to aid the identification of the species of this diverse genus, which occasionally may be difficult to identify to the species-level, due to the lack of reference collections for most countries within its distribution range, and to the presence of intra-specific variation and secondary sexual characters. It is expected that this on-line key will facilitate future periodic updates, and will benefit all those persons interested in identifying these taxa. The present paper also includes the description of *Megalostomis juanenrique* **sp. n.**, a new species from Paraguay. In addition, *Megalostomis gigas* Lacordaire, and *Megalostomis robustipes* Monrós are newly cited for the fauna of Paraguay. The online interactive Lucid key is available at http://keys.lucidcentral.org/keys/v3/megalostomis. Offline Lucid data files in LIF and SDD formats are also available at doi: 10.3897/zookeys.425.7631.app1 and doi: 10.3897/zookeys.425.7631.app2.

## Introduction

South America harbors a high diversity of Clytrini. *Megalostomis* Chevrolat, 1836 is the largest genus within the Neotropical subtribe Megalostomina, its species are phytophagous in the adult stage, primarily leaf and flower feeders. As larvae, they are case-bearers, some species are known to live in ant nests (Riley et al. 2002). Agrain and Roig-Juñent (2011) recently tested the phylogenetic relationships among its species, proposing two species groups with distinct morphological and biogeographical characteristics. More recently, Agrain (2013) provided a taxonomic review of the genus including all its species. Both former works anticipated that the study of specimens from certain countries would surely yield some taxonomic novelties. This was confirmed when studying a small lot of specimens collected by J. M. Viana, over thirty years ago, in Paraguay. The latter not only corroborates the need for further field and curatorial work for this group, but also inspired the need for having an online key that can be easily improved, corrected and expanded in short time and in one place, as our knowledge of these taxa is progressively enriched. Therefore, the main goal of this contribution is to release an interactive multi-entry key to all known species of the genus *Megalostomis* Chevrolat (43 including the new one). It is expected that this type of key will be more valued by the community than traditional printed keys, granting perpetuity of the key’s life, acknowledging the original and subsequent authors (Penev et al. 2009). For more information concerning Lucid keys visit http://www.lucidcentral.org.

## Materials and methods

### Specimens studied

The material examined during this work came from Agr. Eng. Juan Enrique Barriga-Tuñon’s personal Collection (JEBC).

### Depository of types

IADIZA Instituto Argentino de Investigaciones de las Zonas Áridas, Mendoza, Argentina.

INBP Museo Nacional de la Historia Natural del Paraguay, San Lorenzo, Paraguay.

JEBC Juan E. Barriga T. Collection, Curicó, Chile.

MACN Museo Argentina de Ciencias Naturales, Buenos Aires, Argentina.

### Dissections of adults

Specimens were soaked in NH_3_ for ten minutes to relax their anatomical structures. To study the male genitalia, the aedeagus was removed through the pygidium and cleared in 10% hot KOH for five minutes (for the female the whole abdomen was removed). After the KOH clearing process, the aedeagus was washed in 80% ethanol and then glycerin was injected through the basal foramen, using a needle and the help of curved dissecting forceps, in order to evert the internal sac. Upon completion of the examination, the genitalia and other dissected parts were placed in a plastic microvial with glycerin, and pinned directly beneath each specimen.

### Character nomenclature

Terminology follows Agrain 2013 (and references therein).

### Illustrations and photography

All photographs were taken by the author. The source photos were taken at multiple focal planes (at least five) and then combined into a single image (with all features in focus) with the free software CombineZP (http://www.hadleyweb.pwp.blueyonder.co.uk/CZP/News.htm). Images for this study were taken with a Canon PowerShot S50 digital camera, mounted on a Leica S6E stereomicroscope. For the matrix-based key, all illustrations of characters and their states were taken from Agrain and Roig-Juñent (2011) supplementary material, while all species plates were modified from Agrain (2013), except for the illustration of *Megalostomis subnitida* Guérin [modified from Guérin (1952)], and *Megalostomis monrosi* Medvedev [modified from Medvedev 1998].

### Creation of the multi-entry key

The multi-entry interactive key was created by exporting the nexus data matrix used in Agrain and Roig-Juñent (2011), to DELTA software (http://delta-intkey.com). All taxa, and characters with their respective states were imported from .dlt to .lif file format into Lucid builder 3.3 (http://www.lucidcentral.org), by use of Lucid translator (http://www.lucidcentral.com/en-us/software/lucid3/lucidbuilder/lucidtranslator.aspx). Within Lucid builder 3.3 (free edition), pictures for most character states, species plates, country level occurrence data (taken from Agrain 2013 checklist), and a few autapomorphies that were not in the original data matrix were added in order to maximize the probability of precise identification. Online interactive Lucid key is available at http://keys.lucidcentral.org/keys/v3/megalostomis. Lucid data files are provided in appendices as follows: Suppl. material 1: Lucid Interchange Format version 3 (LIF3) file, and Suppl. material 2: SDD (Structure of Descriptive Data) Schema. Also a copy of the deployed key (LKC4), containing high resolution pictures can be obtained from the author’s website: http://fedeagrain.wordpress.com/lucid-keys/ as a zip file, this is the equivalent to a CD-ROM version, for off-line (local) use, once decompressed you will need Lucid Player 3.3 installed on your system to visualize it. This player is multi-platform and is freely available at http://www.lucidcentral.com/en-us/software/lucid3/lucidplayer.aspx.

## Results

### Taxonomy

*Megalostomis* is easily diagnosable from Cryptocephalinae by the following characters: body length 6–14 mm, width 4–10 mm; prothorax without lateral antennal grooves; tarsal claws simple; prosternum evident between procoxae; procoxae globose; eyes strongly emarginate; and dorsal plate of aedeagus with straight margin. The latter two characteristics were found to be synapomorphies for the genus according to Agrain and Roig-Juñent 2011.

#### Megalostomis juanenrique sp. n.

Taxon classificationAnimaliaColeopteraChrysomelidae

http://zoobank.org/A5DD6649-D388-41FE-BDF4-102FECBF81FD

Figs 1 – 3

##### Type locality.

**PARAGUAY**, San Pedro: Cororō (23.439011°S, 56.501807°W).

##### Type specimens.

*Holotype*: male, pinned. Original labels: “White label (handwritten): Paraguay-San Pe /dro-Córoro/ M. Viana XI_1983_, Blue label (printed): ex Coleccion/ M. VIANA/ ARG 006244, White label (printed): Coleccion / J. E. BARRIGA / CHILE 067786”. Red label (printed): *Megalostomis juanenrique* sp. n. / Holotype/ Des. Agrain F. A. 2014. IADIZA. *Allotype*: female, pinned, with genitalia in a separate microvial. Original labels: “White label (handwritten): Paraguay.S.P./ Córoro/ M. Viana 1979, Blue label (printed): ex Coleccion/ M. VIANA/ ARG 006337, White label (printed): Coleccion / J. E. BARRIGA / CHILE 067005”. Red label (printed): *Megalostomis juanenrique* sp. n. / Allotype/ Des. Agrain F. A. 2014. IADIZA. *Paratype*: male, pinned, with genitalia in a separate microvial. Original labels: “White label (printed): PARAGUAY/ Córoro/ dic 1983/ leg. M. Viana, White label (printed): Coleccion / J. E. BARRIGA / CHILE 138773”. INBP. *Paratype*: male, pinned. Original labels: “White label (handwritten): Paraguay-S P/ Córoro/ M. Viana _1976, Blue label (printed): ex Coleccion/ M. VIANA/ ARG 006362, White label (printed): Coleccion / J. E. BARRIGA / CHILE 063063”. MACN. *Paratype*: male, pinned. Original labels: “White label (handwritten): Paraguay-San/ Pedro-Córoro/ XI_1985_ M. Viana, Blue label (printed): ex Coleccion/ M. VIANA/ ARG 006238, White label (printed): Coleccion / J. E. BARRIGA / CHILE 069889”. JEBC. *Paratype*: male, pinned, with genitalia in a separate microvial. Original labels: “White label (handwritten): Paraguay. S.P./ Córoro/ M. Viana 1976, Blue label (printed): ex Coleccion/ M. VIANA/ ARG 006406, White label (printed): Coleccion / J. E. BARRIGA / CHILE 067050”. JEBC. *Paratype*: male, pinned, with genitalia in a separate microvial. Original labels: “White label (handwritten): Paraguay. S.P./ Córoro/ M. Viana 1976, Blue label (printed): ex Coleccion/ M. VIANA/ ARG 006270, White label (printed): Coleccion / J. E. BARRIGA / CHILE 057918”. JEBC. All paratypes with my label: Red label (printed): *Megalostomis juanenrique* sp. n./ Paratype/ Des. Agrain F. A. 2014.

##### Diagnosis.

This new species belongs to the *Megalostomis grossa* species group as defined by Agrain and Roig-Juñent (2011). Characters of this species group exhibited by this species are: uniform antennal coloration, presence of a longitudinal carina at interocular space, and pronotal disc pilosity limited to or denser at its margins; even if this species shares some characters with its relatives (*e.g.* the hypertrophied eye stalk with *Megalostomis grandis* (Forsberg), it can be easily separated from this and other related species by several characters: male mandibles exceeding length of clypeus, head wider than long, subrectangular pronotal disc longer than high, with its lateral margins visible from above, central dorsal plate of kotpresse (female) with three arms, apical margin of dorsal plate of aedeagus straight, among others. As a rule, for this genus, the best way to identify the females (without dissecting them) is by comparing the coloration pattern with their respective males.

##### Body length.

11.3–12.5 mm, width: 6.3–7.2 mm.

##### Male

**Figure 1.**
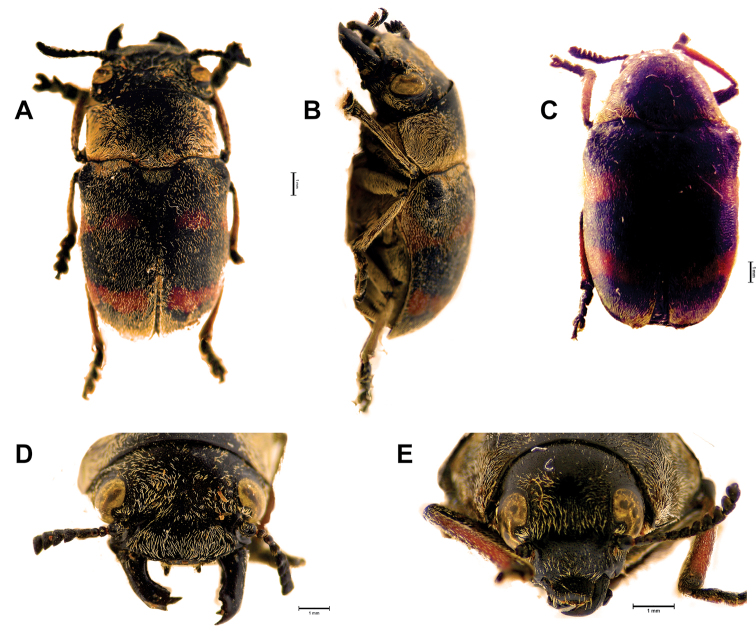
*Megalostomis juanenrique* sp. n. **A** Habitus dorsal (male) **B** Habitus lateral (male) **C** Habitus dorsal (female) **D** Head of male (frontal view) **E** Head of female (frontal view).

**Figure 2.**
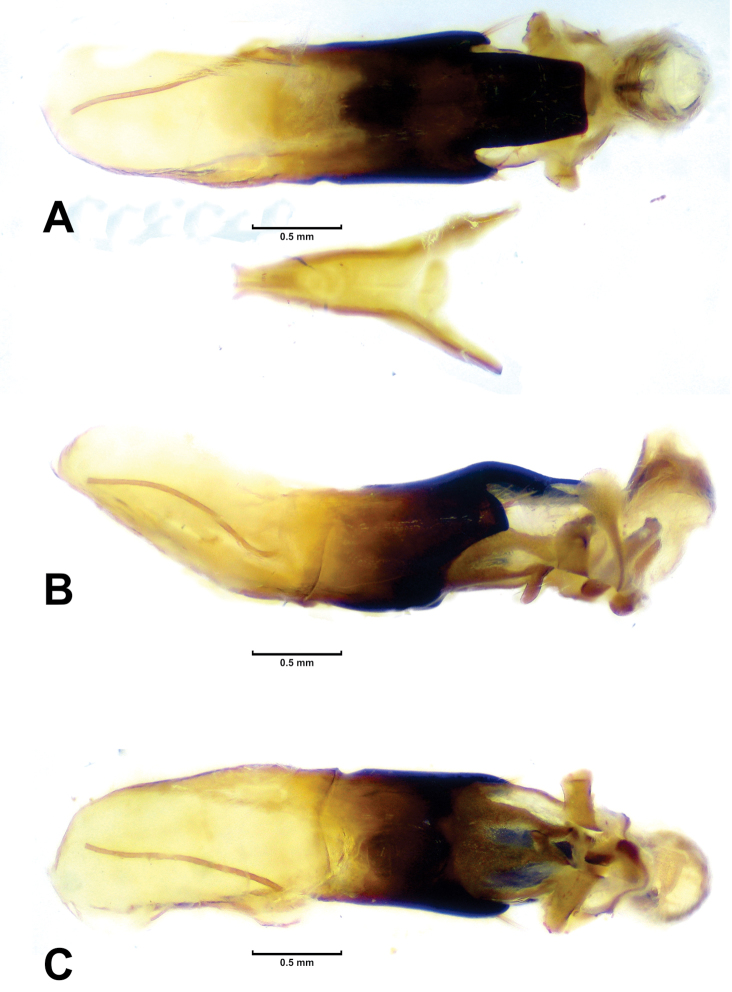
*Megalostomis juanenrique* sp. n. **A–C** Male aedeagus and internal sac: **A** Dorsal view **B** Lateral view **C** Ventral view.

(Figs 1A–B, D, 2). *Coloration pattern*: elytra reddish, with three interspaced sub-transverse black bands, all reaching lateral margin of elytra, basal band reaching humeral carina, and apical band reaching elytral apex. *Head*: anterior surface smooth, with central protuberance at upper region of clypeus, mostly glabrous with disperse pubescence; inter-ocular space with thin longitudinal carina, with a pair of slightly depressed sub-adjacent areas on either side; internal margin of eyes with strongly marked carina, posterior side of eye with salient post-ocular protuberance, next to a marked furrow. Mandibles asymmetric; apex of mandibles with a series of peaks (molariform area) surrounding a depressed area that fits into the right mandible; right mandible curved inward, mandibular apex with a very sharp tooth on its underside; left mandible straight, with sharp bifurcated teeth; with common intra-specific variation in size among examined series. Clypeal margin, straight, without auricular appendix. Antennae: as long as pronotum, black; scape robust; serrate beyond fourth antennomere, eleventh antennomere with two marginal excavations that delimit central lobe. *Thorax*: pronotal disc: longer than high, sub-rectangular, lateral margins visible from above; posterior projection short; median lateral region with slight transverse constriction; punctation weak and regular; pronotal disc pilosity limited to its margins; scutellum wider than long, with posterior margins curved, covered with white dense pubescence. *Elytra*: apical margin projected, surpassing pygidium; elytral humeral region wider than pronotal base, gradually narrowing toward apex; diffuse punctation, denser than that of pronotum; elytral margin narrow, enlarged in humeral region; humeral carina rounded, apex with transverse mark. *Genitalia*: (Fig. 2A–C) apex of dorsal plate of median lobe as wide as its base, with anterior margin straight; lateral arms of median lobe short, with setae; dorsal sclerite of internal sac (not everted) upward-directed, forming a wing-shaped structure in dorsal view; ejaculatory guide with ventral keel; sperm transfer structure with campanulate sclerite.

##### Female

**Figure 3.**
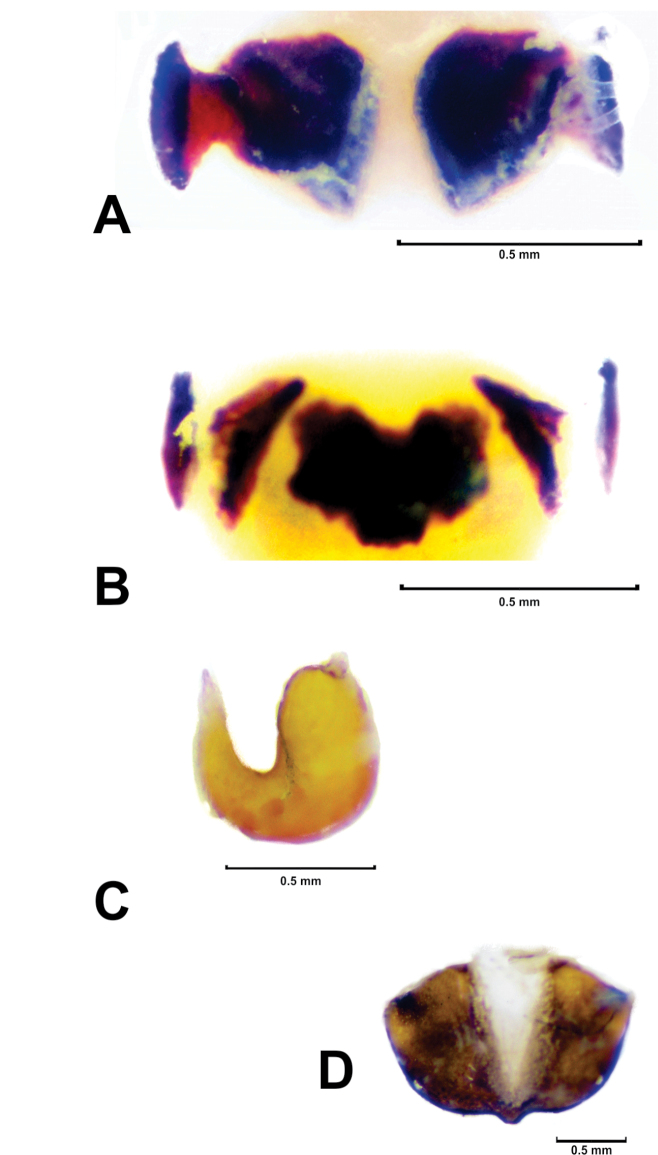
*Megalostomis juanenrique* sp. n. **A** Kotpresse ventral sclerite **B** Kotpresse central dorsal plate and dorsal apodemes **C** Spermathecal capsule **D** Sternite VIII.

(Figs 1C, E, 3). *Coloration pattern*: same as male. *Head*: smaller than that of male; mandibles compact and short. *Antennae*: same as male, but shorter than pronotum (with smaller antennomeres). *Thorax*: pronotum with sides slightly more curved than in male. *Abdomen*: sternites same as in male, fifth sternite excavate. *Pygidium*: with apical excavation and, apical transverse depressed area. *Rectal sclerites*: dorsal rectal sclerites (Fig. 3A) represented only by dorsal (subquadrate) apodemes, and central dorsal plate. Central dorsal plate (Fig. 3B) subquadrangular with three arms; ventral rectal sclerites with very short apodemes. *Spermathecal capsule*: (Fig. 3C) U-shaped, distal part more than 2× longer than proximal part, proximal part longer than base; angle formed between basal and apical regions of spermathecal capsule less than 45°; apex of spermathecal capsule long, with sharp tip. Eighth sternite with central tooth (Fig. 3D).

##### Etymology.

Specific name is treated as a noun in apposition (ICZN 1999, Art. 34.2.1), it is dedicated to the distinguished Chilean coleopterist Agr. Eng. Juan Enrique Barriga-Tuñon, who generously received me in his outstanding insect collection.

### Species newly recorded for Paraguay

***Megalostomis gigas* Lacordaire**

One specimen from: **PARAGUAY**, San Pedro: Cororō (23.439011°S, 56.501807°W). February 1979, Leg. J. M. Viana. JEBC.

***Megalostomis robustipes* Monrós**

One specimen from: **PARAGUAY**, Dep. Cordillera: Pirareta, (25.483333°S, 56.933333°W). 26–31 August, 2011. Leg. U. Drechsel. IADIZA.

### An interactive multi-entry key to the species of *Megalostomis*

The main benefit of the interactive multi-entry key is that the user needs to follow fewer options compared with traditional dichotomous keys, and these options can be freely chosen; this generally leads to more effective identification. More important, both experienced and inexperienced users are more likely to succeed when identifying problematic species (Drinkwater 2009). The software used to visualizes the key allows “filtered” and “ranked” options when determining a specimen. The first option will eliminate taxa that do not coincide with the chosen character states; while the latter option will keep all taxa, but listing them in priority order in accordance with their agreement (expressed in percentages) with the chosen character states. This latter option is likely to reduce erroneous identifications. According to the score analyzer of the software, the key presented in this paper has at most 23 state differences to diagnose the species of *Megalostomis*. Therefore, when using the key, it is worthwhile to keep adding character states even if the identification is finished, or to start the identification over again, selecting a different set of characters in order to ensure accurate identification. Users of this key should keep in mind that this was created directly from a phylogenetic dataset, so it has more character to choose from than a traditional key which is focused on diagnostic characters. As a final advice, even if the key includes country level occurrences for each species, it is better to start the identification by using morphological characters, including country level occurrences only to confirm identity, since distribution data is lacking for several countries.

## Supplementary Material

XML Treatment for Megalostomis juanenrique

## Supplementary materials

Supplementary material 1 Key to the species of *Megalostomis* v.1.0, LIF3 file doi: 10.3897/zookeys.425.7631.app1 Data type: Lucid Interchange Format version 3 (LIF3), file: Key to the species of *Megalostomis* v.1.0. Explanation note: This XML-based format allows exchange of the key with other key developers. This dataset is made available under the Open Database License (http://opendatacommons.org/licenses/odbl/1.0/). The Open Database License (ODbL) is a license agreement intended to allow users to freely share, modify, and use this Dataset while maintaining this same freedom for others, provided that the original source and author(s) are credited.Federico A. Agrain

Supplementary material 2 Key to the species of *Megalostomis* v.1.0, SDD file doi: 10.3897/zookeys.425.7631.app2 Data type: Lucid SDD file: Key to the species of *Megalostomis* v.1.0. Explanation note: This is a XML-based file structured using the internationally agreed SDD (Structure of Descriptive Data) Schema. This SDD file may be used to exchange the Lucid key with other SDD-compliant applications. This dataset is made available under the Open Database License (http://opendatacommons.org/licenses/odbl/1.0/). The Open Database License (ODbL) is a license agreement intended to allow users to freely share, modify, and use this Dataset while maintaining this same freedom for others, provided that the original source and author(s) are credited.Federico A. Agrain
